# Lesbian, gay, bisexual, transgender, queer and intersex (LGBTQI+) healthcare in Singapore: perspectives of non-governmental organisations and clinical year medical students

**DOI:** 10.1080/10872981.2023.2172744

**Published:** 2023-02-06

**Authors:** Caitlin A O’Hara, Xiang Lin Foon, Jared CK Ng, Chen Seong Wong, Francine YC Wang, Clara YR Tan, Yi Ting Cheah, Konstadina Griva, Joanne SY Yoong, Rayner KJ Tan

**Affiliations:** a Yong Loo Lin School of Medicine, National University of Singapore, Singapore; b National Center for Infectious Diseases, National University of Singapore, Singapore; c Office of Research, Lee Kong Chian School of Medicine, Nanyang Technological University, Singapore; d Research for Impact, Singapore; e Saw Swee Hock School of Public Health, National University of Singapore, Singapore; f University of North Carolina Project-China, Guangzhou, China

**Keywords:** LGBTQ+ health, medical education, healthcare stigma, healthcare discrimination, medical training

## Abstract

**Purpose:**

International studies document that lesbian, gay, bisexual, transgender, queer and intersex (LGBTQI+) patients face significant health disparities. Studies exploring the attitudes, knowledge, preparedness and comfort levels of healthcare students towards LGBTQI+ health have been conducted in the United States, United Kingdom and Malaysia. This study aims to investigate stigma in healthcare for LGBTQI+ patients in Singapore, and possible upstream factors within medical education.

**Methods:**

This mixed-methods study adopts a convergent parallel design. The Health Stigma and Discrimination Framework was referenced to devise in-depth interviews with representatives from 13 LGBTQI-affirming non-governmental organisations, analysed through thematic analysis. 320 clinical medical students were surveyed about attitudes, knowledge, comfort, preparedness, and perceived importance of/towards LGBTQI+ health, analysed via descriptive statistics and multivariate regression.

**Results:**

Prevailing stigma in Singaporean society against LGBTQI+ individuals is exacerbated in healthcare settings. Doctors were cited as unfamiliar or uncomfortable with LGBTQI+ health, possibly from lack of training. Among medical students surveyed, the median composite attitudes, comfort and preparedness index was 3.30 (Interquartile Range (IQR) = 0.50), 3.17 (IQR = 0.83), 2.50 (IQR = 1.00) respectively. Only 12.19% of students answered all 11 true-false questions about LGBTQI+ health correctly.

**Conclusion:**

Medical students in Singapore have scored sub-optimally in their knowledge and preparedness towards LGBTQI+ health, while interpersonal and structural stigma in healthcare towards LGBTQI+ people in Singapore negatively affects health and wellbeing. These findings are an impetus to improve medical training in this area. High scores among medical students in attitudes, comfort and perceived importance of LGBTQI+ topics demonstrate that there is space for LGBTQI+ health in the local medical education curriculum. Curricular interventions can prioritise content knowledge, communication skills and sensitivity.

## Introduction

Stigma against lesbian, gay, bisexual, transgender, queer and intersex (LGBTQI+) individuals has been comprehensively documented in international literature [1–3]. Stigma is a social construction through which individuals are devalued on the basis of a ‘mark’; stigma and its ensuant discriminatory processes can adversely influence health through various mechanisms [4]. Stigma manifests in several forms which can occur simultaneously: anticipated stigma (foreseen to be acted upon oneself by others), internalised stigma (negative beliefs one adopts about oneself due to external influences), or enacted stigma at the interpersonal (e.g., overt or covert behaviours between individuals) and structural (e.g., discriminatory policies and laws, or societal norms) levels [4,5].

Stigma against LGBTQI+ individuals also exists within healthcare settings [6–8], which can contribute to known healthcare disparities present within this population [9,10]. A thorough understanding of how stigma operates throughout healthcare is key in improving the system through structural means (e.g., organizational culture and quality of care standards), interpersonal means (e.g., doctor-patient relationships and attitudes of healthcare providers) and intrapersonal means (e.g., self-stigma and care avoidance or non-disclosure) [11]. Healthcare access is the ‘opportunity to identify healthcare needs, to seek healthcare services, to reach, to obtain or use health care services and to actually have the need for services fulfilled’; accessibility encompasses appropriateness, acceptability and approachability in addition to affordability and availability [12]. LGBTQI+ individuals are at risk of poorer healthcare access and health outcomes globally [13], and in Singapore this matters especially due to prevailing stigma towards LGBTQI+ individuals, as well as services and policies that do not affirm LGBTQI+ individuals or families [14]. In Singapore, LGBTQI+ individuals are at increased risk of experiencing mental health problems, engaging in substance use, having difficulty accessing gender-affirming care, and encountering discrimination and violence [15–18]. It is imperative that healthcare in Singapore is accessible to the LGBTQI+ population to address these needs, in ways that cater to their unique identities.

A key determinant of healthcare delivery are healthcare personnel themselves. Medical school is where both knowledge and professionalism-related foundations for a doctor’s future clinical practice are set [19]; medical education has immense potential to shape LGBTQI+ healthcare for the better [20]. Studies have previously been done in Malaysia, the United States, the United Kingdom and Canada to examine the knowledge, attitudes, preparedness, comfort levels and perceptions of curricular coverage among medical students towards LGBTQI+ health [21–25]. No similar studies have been done in Singapore.

Inclusion of LGBTQI+ bias reduction and training programmes has been shown to increase knowledge of health issues, and improve attitudes and comfort towards LGBTQI+ patients [26,27]. Health disparities among LGBTQI+ patients can be partially attributable to a lack of medical training, whether through inadequate treatment of conditions in LGBTQI+ patients, through discrimination, or through deterring healthcare-seeking behaviour [28]. Training in LGBTQI+ health would be especially beneficial in a Singapore context wherein LGBTQI+ health disparities are present, and LGBTQI+ health is not presently well-addressed in medical education syllabuses [29–31].

With the Singapore Medical Council’s Ethical Code and Ethical Guidelines explicitly stating that discrimination or bias against patients on the basis of gender or sexual orientation is disallowed [32], it would be pertinent to explore how clinical medical students in Singapore relate to LGBTQI+ health as they would have both gone through key medical training, and soon be practising medicine. Additionally, no studies have documented how stigma manifests towards LGBTQI+ individuals in Singapore within healthcare. The aims of this study are thus twofold. Firstly, this study aims to investigate the manifestations of stigma against the LBGTQI+ population in the Singapore healthcare setting from an organisational perspective. Secondly, this study aims to investigate possible upstream factors among Singaporean medical students which could influence both the cause and amelioration of healthcare-associated stigma.

## Methods

This study adopts a convergent parallel design [33], comprising in-depth interviews regarding stigma against LGBTQI+ individuals in healthcare in Singapore, and a survey of medical students’ knowledge, attitudes, preparedness, comfort levels and perceived importance with regards to LGBTQI+ health issues. Ethics approval was obtained from the National University of Singapore Institutional Review Board (NUS-IRB-2020-351). Both quantitative and qualitative arms were completed in accordance with the Declaration of Helsinki as revised in 2013.

### Qualitative component

In our qualitative work, we adopted an interpretivist epistemological approach, which is premised on a relativist ontology and centers human interpretation as the starting point for the construction of knowledge in the social world [34]. Thirteen in-depth interviews were conducted with representatives from LGBTQI-affirming non-governmental organisations in Singapore. A list of all key LGBTQI+ organisations in Singapore was first prepared, comprising both government-registered entities and informal community groups. Interviewees were recruited via purposive sampling from these organisations to represent various subpopulations within the LGBTQI+ community. To be an interviewee, one had to self-identify as a representative of an organisation serving the interests of the Singaporean LGBTQI+ community, and be at least 18 years old. This was aligned with our aim of the study in understanding how members of the community, with both lived experiences of discrimination and who have assisted individuals with similar experiences, construct and navigate stigma and discrimination in health and healthcare. Organisation representatives were chosen as interviewees given the fear amongst the LGBTQI+ population of being outed if interviewed individually on this topic. Moreover, we posited that organisation representatives would be better equipped to provide insight on institutional-level recommendations to improve healthcare-related stigma.

The organisations in the sample were representative of a large portion of the landscape of LGBTQI+ organisations in Singapore, and comprised both officially government-registered entities, as well as informal community organisations doing groundwork with the local LGBTQI+ community. This spread of organisations lent the sample great information power [35]. To this end, saturation was not used as a guiding paradigm to determine the sample size of our study, and we determined that representatives from these 13 organisations would enable us to reach a multitude of perspectives reflecting the experiences of the wider LGBTQI+ community in Singapore. Table 1 summarises the characteristics of participants and their organisations. Interviews were conducted over the online teleconferencing software, Zoom. Informed consent for the interviews was obtained verbally in lieu of documented consent.

**Table 1.** Overview of organisations interviewed in qualitative component of the study.

Organisation’s focus	Type of organisation	Role in organisation	Reference number
LBTQ women	Non-governmental organisation	Programme manager	001
General LGBTQI+ community	Non-governmental organisation	Programme leader	002
Sexual health of the LGBTQI+ community	Non-governmental organisation	Programme leader	003
Religious LGBTQI+ individuals	Queer-affirming religious organisation	Programme leader	004
General LGBTQI+ community	Queer-affirming commercial entity	Programme leader	005
Trauma survivors who are LGBTQI+	Queer-affirming social enterprise for trauma recovery	Programme leader	006
Transgender community	Non-governmental organisation	Community worker	007
Transgender community	Non-governmental organisation	Member	008
General LGBTQI+ community	Community group	Member	009
University-based LGBTQI+ community	University-based organisation	Programme leader	010
General LGBTQI+ community	Non-governmental organisation	Member	011
Bisexual individuals	Community group	Programme leader	012
LGBTQI+ youth	Queer-affirming youth community group	Programme leader	013

Semi-structured interviews were conducted, with the interview guide devised based on the Health Stigma and Discrimination Framework [36]. A copy is available in Supplementary Appendix SA1. Where applicable, interviewees were invited to comment with respect to the unique subgroup of LGBTQI+ individuals that their organisation served, and to draw examples from their organisation’s clients or community members. Each interview lasted approximately one hour. Interviews were conducted in English, audio-recorded, de-identified and transcribed verbatim prior to coding. Participants were reimbursed with a 50 Singapore dollar shopping voucher. Interviews were conducted by CAO, FXL and NCKJ, who had previously conducted and were trained in qualitative research.

The Health Stigma and Discrimination Framework [36] was used to deductively code the transcripts. While the original framework provided the initial codes for coding, new codes were generated throughout the process of analysis, and were then inductively grouped to generate the themes that were reported in this manuscript. The coding frame was devised iteratively, with study team members first coding several transcripts collectively and reaching a consensus coding frame, prior to independently coding the remaining transcripts. A total of four coders (CAO, FXL, NCKJ, RKJT) were involved in the coding and thematic analysis process. Discrepancies in coding were resolved based on regular coding meetings where differences were brought up, discussed at length, and subsequently agreed upon by all coders. Salient themes were extracted based on the final codes devised.

Reflexivity is crucial for generating trustworthy insights in qualitative research [37], and to this end we consciously reflected on several aspects of the researcher’s role and positionality through the course of the study. The principal investigator and members of the research team, while primarily members of an academic institution, also identify and engage regularly with members of the LGBTQI+ community in Singapore through community work. As such, this shared affiliation and at times identity with study participants created opportunities for greater rapport and trust. Nevertheless, the study team are also wary that our shared identities and experiences meant the risk of privileging the researchers’ narratives over those of participants. To mitigate this, the study team met regularly to discuss such dynamics and reflect on the process.

### Quantitative component

Stratified sampling was employed to recruit 320 clinical medical students from the three local medical schools in Singapore from April to May 2021. Given that there were 456 medical graduates in Singapore in 2021 [38], we estimate that a single cohort across all three medical schools would have 500 students. As each medical school has three clinical years of study, there would be about 1500 clinical year students in Singapore at the point of this study. With this population size, a sample size of 306 was required to achieve 95% confidence and a 5% margin of error.

The questionnaire was created as per the process described by Artino et al [39]. A literature search was conducted. The survey instruments were first derived from existing studies, adapted to better suit the local context, and subsequently reviewed by local experts. The attitudes measure was adapted from the Attitudes Towards LGBT Persons Scale-Malaysia (ATLPS-M) used by Foong and colleagues [25], who validated in a Malaysian context a modified version of the Attitudes Towards LGBT Persons Scale (ATLPS) developed originally by Sanchez [40]. As the socio-cultural contexts of Malaysia and Singapore are similar, our study adapted the ATLPS-M. Knowledge was assessed through 11 true/false statements to which respondents could select ‘true’, ‘false’ or ‘I don’t know’. These statements were chosen with reference to studies done among medical students and healthcare providers in the United States [24,41]. The difficulty of and answers for the statements were validated as appropriate for clinical-year medical students among medical specialists in Singapore from obstetrics and gynaecology, psychiatry, infectious diseases and endocrinology. These medical specialists were contacted directly via the study team as they had been known through word-of-mouth within the LGBTQI+ community to be LGBTQI-affirming in their own clinical practice. Participants were allowed to choose ‘I don’t know’ to prevent correct answers from being chosen through guessed attempts. All ‘I don’t know’ responses were coded as incorrect to reflect the presence of a knowledge gap.

The survey was first piloted among a smaller group of 15 students to ensure the questions were phrased in a way that could be understood by the general local clinical student, and that the time taken to complete the survey was adequate. Participants were required to report being at least 21 years old, and in their clinical phase of education. Upon completing the survey, participants could leave an email address to receive an SGD 5.00 Grab Ride transportation voucher. Informed consent was obtained via checking a box to agree to participate. The median time to complete each survey was approximately 7 minutes. Respondents were asked to fill in demographic information about themselves and their relationship to the LGBTQI+ community, then to answer questions concerning their attitudes, knowledge levels, comfort levels, preparedness levels and perceived importance with respect to LGBTQI+ health. The full questionnaire is in Supplementary Appendix SA2.

Self-reported preparedness and comfort was measured with respect to six statements concerning the care of LGBTQI+ patients. These statements mirrored that in a study done among medical students in the United States [24]. Participants were additionally asked how important they perceived 17 topics concerning LGBTQI+ health to be, adapted from a similar study in the United States [21] and approved by the aforementioned Singaporean medical specialists. Finally, participants rated how adequately they perceived the coverage of LGBTQI+ health content in their medical school syllabus to be, and were given the option to leave free text comments.

### Statistical analysis

Statistical analysis was performed on STATA version 17. Descriptive statistics were employed to examine the composite or total scores for attitudes, knowledge, preparedness, comfort, and perceived importance. Cronbach’s alpha was measured for all composite indices to ensure internal reliability. Multivariate analysis was performed to examine associations between the outcome variables and socio-demographics. We conducted ordinary least squares (OLS) regression (or linear regression) for the outcome variables of attitudes, preparedness, comfort and perceived importance. Diagnostic tests were performed to assess the suitability of an OLS regression approach. We omitted the model for the outcome of knowledge as the residuals of the model were not normally distributed and did not meet the assumptions to perform a multivariate linear regression. Statistical significance was set at *p* < 0.05.

## Results

Table 2 summarises the socio-demographics of the sample. Cronbach’s alpha was 0.62, 0.67, 0.88, 0.90 and 0.95 for attitudes, knowledge, comfort, preparedness and perceived importance respectively. Thus, each composite measure was internally reliable to an at least acceptable degree [42].

**Table 2.** Descriptive statistics from quantitative component of the study (*n* = 320).

Demographic Variables	n/Mean	Percentage/S.D.
**Gender**		
Male	151	47.19
Female	167	52.19
Others	2	0.63
**Medical school**		
Medical school 1	191	59.69
Medical school 2	98	30.63
Medical school 3	31	9.69
**Year of Study**		
First clinical year	138	43.13
Second clinical year	115	35.94
Third clinical year	67	20.94
**Religion**		
Agnosticism/atheism/no religion	143	44.69
Christianity	118	36.88
Islam	3	0.94
Buddhism	36	11.25
Hinduism	14	4.38
Sikhism	1	0.31
Taoism	5	1.56
**Identifies as LGBTQI+**		
No	282	88.13
Yes	38	11.88
**Knows of anyone who is LGBTQI+**		
No	41	12.81
Yes	279	87.19
**Closeness to LGBTQI+ contact1(*n* = 279)2**	7	3, 8
**Relationship to LGBTQI+ contact (*n* = 279)2**		
Acquaintance	40	14.34
Colleague	23	8.24
Friend	194	69.53
Partner	15	5.38
Relative	7	2.51
**Identifies as an ally (*n* = 282)3**		
Yes	137	48.58
No	48	17.02
Unsure	70	24.82
Prefers not to say	27	9.57
**Composite attitudes score4**	3.21	0.367
**Composite knowledge score4**	7.91	2.27
**Composite preparedness score4**	2.50	0.732
**Composite comfort score4**	3.20	0.571
**Composite perceived importance score4**	3.38	0.541
**Perception of adequacy for curricular coverage of LGBTQI+ health issues4**	1.74	0.738

^1^– Median with composite values.

^2^– only answered by those who know someone who is LGBTQI+.

^3^– only answered by those who do not identify as LGBTQI+ themselves.

^4^– reporting mean and standard deviation instead of frequency and percentage.

The interviewees expounded on how stigma manifests towards the LGBTQI+ community in Singapore, both within and outside of healthcare, and across the individual, interpersonal and structural dimensions. While the medical students surveyed had moderately positive attitudes and comfort levels towards LGBTQI+ patients, their levels of knowledge and preparedness towards treating LGBTQI+ health issues were sub-optimal. Interviewees shared about health and social impacts of stigma in the local healthcare setting, along with recommendations on how to reduce stigma. These results are elaborated upon in this section.

### Singapore as an inherently non-LGBTQI-affirming society

Interviewees commented on how stigma against the LGBTQI+ community already exists in Singapore society, which in turn sets the scene for stigma (whether anticipated or enacted) within the healthcare setting and internalised stigma on the part of LGBTQI+ patients themselves. Table 3 depicts a summary of the sub-themes pertaining to pre-existing stigma in Singapore. Structural stigma was highlighted on multiple levels: cultural norms, discriminatory laws, and the lack of protective policies such as anti-discrimination laws.

**Table 3.** Sub-theme 1: *Singapore as a non-queer-affirming society to live in from the outset.*

Subtheme	Quote	Interview reference number
Care avoidance due to perceptions that providers would be discriminatory	*‘So the lack of societal understanding would cultivate a hostile society in general, which leads into a lack of understanding of healthcare providers … we can speculate that they (people who did not disclose their sexuality to doctors in a series of interviews done by the organisation) were either not comfortable due to environment, you know, societal stigma at large, or, and or like the perception that the provider would be discriminatory … ’* *‘The lack of anti-discrimination policies or guidelines, like, you know, like you said, the doctor patient relationship, there is no guideline that outlines the treatment of LGBT persons. So it leaves it up to the individual doctor’s discretion to treat you, however, they want to depending on their bias.’*	001, Representative of an organisation supporting LBTQ women in Singapore
Distrust towards institutions nationwide	*‘.It’s the notion that if you know, you’re LGBTQ, you know, that, like, you, you shouldn’t trust any institution in this country. Yeah, and it’s like, I mean, you, you know, that you shouldn’t trust the school, you know, you shouldn’t trust the healthcare system, you know, you shouldn’t trust the like, You’re, like, wherever you’re seeking employment, whatever. So it’s like, you have to not trust by default. And if you trust by default, you will get hurt. That is just like almost a certainty, I feel.’*	010, Representative of a LGBTQ university student organisation in Singapore
Cisnormativity and heteronormativity in Singapore at large	*‘Yeah, I think like, whether it’s healthcare or like society, in general, there’s a very big, like, gap in, like the awareness towards queer people and queer issues. So like, with that ignorance, like people don’t address like trans people with the proper terms, or like, there is, like, there is heteronormativity, right. So like people, like, just assume that queer people don’t exist, or like queer relationships, queer love doesn’t exist’*	013, Representative from a community group that shares stories of LGBTQI+ youth in Singapore
Internalised stigma as a result of national laws in place	*Okay, let’s let’s talk about our clients. Like, honestly, for them to seek help is incredibly difficult, because 90% of our clients are gay men, who are using drugs and engaging in sex when they are using. Half of them are already HIV positive. So that’s three different laws that they are breaking, like the 377A thing by having gay sex, half of them are HIV positive, they don’t always consistently disclose their status to others before they have sex, and thirdly they are using drugs, right. So there’s three laws that they’re breaking. So when they seek help there’s a lot of shame, guilt and fear of like, persecution, legal implications.*	006, Representative of an organisation that supports trauma survivors
Care providers not being publicly LGBTQ±affirming for fear of persecution	*‘About how the transgender community accesses health care, a lot of time it’s through recommendations .a lot of word of mouth … A lot of hospitals, a lot of doctors don’t want to openly advertise that they do work with transgender clients … Even doctors then are afraid of being stigmatized by the hospital. … (We have heard of a) doctor who almost lost their license because a parent or somebody complained to the hospital, and the hospital then came down quite hard on the doctor. So again, this doctor is like, currently practicing still, but very underground.’*	007, Representative of an organisation supporting the transgender community in Singapore

This stigma may have tangible consequences. Firstly, it may lead to internalised stigma and care avoidance in LGBTQI+ patients. Secondly, healthcare providers may adopt stigmatising attitudes. Thirdly, with a general climate that is not non-LGBTQI-affirming, healthcare institutions and professionals may become disinclined to be openly LGBTQI-affirming for fear of negative impressions among the wider public. This makes care-seeking difficult to navigate for LGBTQI+ patients.

### Healthcare settings compound background stigma in Singapore

Table 4 details the subtheme of stigma in healthcare settings. Interviewees remarked at how the background stigma in Singapore was compounded within healthcare, citing examples of healthcare providers asking questions that were poorly-catered to an LGBTQI+ patient due to heteronormative assumptions. While such stigmatising behaviour of healthcare providers could stem from ignorance or a lack of training, some interviewees shared accounts of more overt discrimination from healthcare providers as well. LGBTQI-related stigma can manifest in multiple healthcare settings, ranging from sexual and reproductive health clinics to the general medical ward.

**Table 4.** Sub-theme 2: *Healthcare settings compound background stigma in Singapore.*

Subtheme	Quote	Interview reference number
Uncertainty about doctor’s attitudes towards LGBTQI+ issues as a barrier to thorough healthcare	*‘Sometimes the question would be like, are you sexually active? … Do you mean sexually, penetrative sex? … You don’t know if the gynae meant sex with men or whether the gynae would discriminate. So a lot of them, they do not disclose their, who do not disclose their identity, do not – cannot - give their doctors a comprehensive view of their life, which impacts the care that they receive.’*	001, Representative of an organisation supporting LBTQ women in Singapore
Provider’s and patient’s religious beliefs as a basis for discrimination	*‘We had a client, one of the counsellors from [redacted], her client said, you know, she went in to consult this doctor on some issue, let slip that she’s lesbian, and for the first time in her life she got a two-hour lecture from a doctor about how, as a lesbian and as a Christian she shouldn’t be doing this … ’*	002, Representative from an organisation supporting the general LGBTQI+ community in Singapore
Providers disregarding the validity of gender-affirming/transgender-specific care	*‘There are those on the extreme end of the spectrum, where they do not know anything about the trans community, they have their certain personal beliefs against the community. And they basically don’t view gender dysphoria or the trans community … they don’t view (it) as a medical need to see us.’*	008, Representative of an organisation supporting transgender individuals in Singapore
Gendered nature of healthcare administration resulting in discomfort for gender-diverse patients	*‘If the medical things sort is like gender specific, and because like legally like, like, most people are recognized by the sex assigned at birth, right. So it could invoke, like a sense of dysphoria, having to like be referred to by the dead name, or like, be referred to, like, the gender pronouns, or like gender terms that do not align with their gender identity. These can be very, like dysphoric, and traumatizing experiences for trans folks try to seek medical attention*	013, Representative from a community group that shares stories of LGBTQI+ youth in Singapore
Lack of research and guidelines for hormone replacement therapy for transgender individuals	*‘(regarding hormone replacement therapy:) The lack of studies done by medical professionals as well, on us, because from what I understand that there just isn’t enough studies done on trans people, you know. For example, when we undergo HRT, you know, does that, because that stops our reproductive reproductive system from functioning, is there a direct relation to to cancer?… when we ask doctors, the doctors, the medical professionals, they, they themselves do not know.’*	008, Representative of an organisation supporting transgender individuals in Singapore

These accounts of poor competence among healthcare providers when dealing with LGBTQI+ health issues was reflected in the low levels of preparedness reported by the medical students surveyed. The median composite preparedness index assessing basic LGBTQI+ health competencies was 2.50 (Interquartile Range (IQR): 1.00, range: 1–4). Comfort scores were however relatively high in contrast, with the median composite comfort index being 3.17 (IQR: 0.83, range: 1–4). Similarly, attitude scores in the sample were relatively high: the median attitudes score was 3.30 (IQR: 0.50, range: 1–4). Multiple linear regression also depicted that medical students belonging to Abrahamic religions had poorer attitudes and comfort levels with regards to LGBTQI+ health. In contrast, students who identified as LGBTQI+ themselves, or who identified as allies, scored better in both comfort and attitudes. These findings are depicted in Table 5.

**Table 5.** Multivariable linear regression models.

Variables	Attitudes		Preparedness		Comfort		Importance	
	Coeff^1^	95% CI^2^	Coeff	95% CI	Coeff	95% CI	Coeff	95% CI
Medical School B (ref= Medical School A)	−0.044	−0.124, 0.035	0.144	−0.033, 0.322	−0.187	−0.151, 0.113	−0.125*	**-0.247, −0.003**
Medical School C (ref= Medical School A)	−0.206**	**-0.328, −0.085**	0.147	−0.123, 0.417	0.044	−0.157, 0.244	0.085	−0.101, 0.371
Year of Study	−0.003	−0.049, 0.043	0.047	−0.054, 0.149	−0.290	−0.105, 0.047	−0.065	−0.135, 0.006
Female (ref = Male)	0.016	−0.057, 0.301	−0.204*	**-0.366, −0.421**	−0.030	−0.150, 0.090	0.176**	**0.064, 0.288**
Others (ref = Male)	−0.148	−0.597, 0.301	−0.733	−1.732, 0.266	−0.140	−0.882, 0.602	0.196	−0.492, 0.884
Abrahamic Religion (i.e., Christianity or Islam)	−0.108*	**-0.192, −0.024**	−0.118	−0.304, 0.677	−0.208**	**-0.346, −0.070**	−0.025	−0.153, 0.103
Knowing someone LGBTQI+	0.058	−0.051, 0.167	0.051	−0.192, 0.293	0.088	−0.092, 0.268	0.177*	**0.009, 0.344**
Identifies as an ally (ref= does not identify)	0.311***	**0.226, 0.381**	0.306**	**0.115, 0.496**	0.326***	**0.184, 0.467**	0.366***	**0.234, 0.497**
Identifies as LGBTQI+ themselves (ref= does not identify)	0.256***	**0.131, 0.381**	0.148	−0.130, 0.426	0.416***	**0.209, 0.622**	0.423***	**0.231, 0.614**

^1^Coefficient.

^2^95% Confidence Interval.

*p < 0.05 **p < 0.01 ***p < 0.001, bold text indicated statistically significant estimates.

Besides stigma enacted by healthcare providers, interviewees shared how administrative systems within healthcare could stigmatise against LGBTQI+ individuals – a form of structural stigma. Another form of structural stigma raised was the lack of research into LGBTQI+ health issues, and a resultant lack of knowledge among doctors.

This low level of familiarity with regards to LGBTQI+ health issues was mirrored in the survey of medical students, wherein 87.81% were unable to answer all the 11 basic true/false questions on LGBTQI+ health correctly. For the true/false question which assessed the difference in risk of suicidal ideation and attempted suicide between LGBTQI+ individuals and cisgender-heterosexual individuals, 25% of respondents reported being unsure of the correct answer. For the true/false question of whether transgender men may need pap smears, 33% of respondents reported being unsure of the correct answer. That such high proportions of students were unsure of the correct answer highlights a lack of familiarity. Accompanying this, 86.25% of the sample either ‘somewhat disagree[d]’ or ‘disagree[d]’ that their medical syllabus adequately covered LGBTQI+ health issues.

### Overlapping, intersecting stigmas and consequences of stigma

Table 6 depicts the subtheme of stigma consequences, including how certain LGBTQI+ patient profiles are affected by overlapping, intersecting stigmas due to multiple identities they hold. Throughout our interviews, respondents articulated negative physical and mental health sequelae of stigma. For instance, internalised and anticipated stigma would lead to avoidance of formal care by patients (which could lead to dangerous consequences from self-medication or delays in diagnosis or treatment); enacted stigma in the form of doctors treating patients based on inherent biases may lead to missed diagnoses; enacted stigma in the form of discriminatory policies and rules would lead to negative mental health repercussions when LGBTQI+ patients are denied healthcare, or negative physical health repercussions when these policies impede access to necessary medical care.

**Table 6.** Sub-theme 3: *Overlapping, intersecting stigmas and consequences of stigma.*

Subtheme	Quote	Interview reference number
Self-medicating at unsafe doses on hormone replacement therapy	*‘We did have a client who, who did self-medication and then for that he has been overdosing on it. Yes, yeah. Because there’s this misconception that, you know, when when you inject … more testosterone, you, you have the misconception that you will transition faster, the result of coming faster … At the same time, it’s very dangerous, because you never know if there are any side effects for having so much, you know, hormones in your body.’*	008, Representative of an organisation supporting the transgender community in Singapore
Biased assumptions from a healthcare provider resulting in missed diagnosis	*‘… As it turned out, this person was presenting COVID symptoms. So, it was something along the – I suppose you could say it was a quick assumption this doctor had made, that you know, sexually active gay men presenting oral symptoms – must be oral STI, right? And then only ordered the oral STI tests, but not COVID.’*	002, Representative from an organisation supporting the general LGBTQI+ community in Singapore
Inability to access gender-affirming care leading to adverse mental health outcomes	*‘With the Ashlee case (a well-known account of a transgender youth who was disallowed from seeking gender-affirming care by her school in Singpaore), we were quite involved in that as well, in terms of … the issues she faced. She mentioned that, like, how it impacted her mental health and led to suicidality. So because of the inability to seek trans health care, the mental health issues come down.’*	001, Representative of an organisation supporting LBTQ women in Singapore
HIV-related laws preventing access to care of non-Singaporean patients	*‘So I have a client, because he’s a foreign student, he didn’t want anyone to know that he has HIV, because of fear that if they will know, then he cannot continue his studies anymore. Um, so he actually didn’t seek help for like, a while. … And finally, when he reappeared again, he looked really, really sick … . the moment he gets treated in Singapore ... means his student pass or student visa will be revoked.’*	003, Representative from an organisation which works towards addressed sexual health in Singapore
Elderly transgender individuals feeling like outcasts and avoiding the healthcare system	*But we do see a lot of cases where, you know, they come and stay in [redacted – organization being interviewed] and they have not sought, they’ve not gone to the doctor, and yes, they’re supposed to have regular follow ups, they don’t go. We have had cases where the person was having eyesight issues, cataract, and just had not gone to the doctor for like, to check on it at all … for the older trans community, they already see themselves as outcasted. … They’ve lived in a society where they they accepted the fact that they are not going to receive any help.*	007, Representative from an organisation that supports the transgender community in Singapore
Language and socioeconomic class as a cause of differential access to knowledge of LGBTQ±affirming services	*‘… With language and socioeconomic class,… say, the English-speaking community, and knowing that “okay, yeah, this doctor is gay-friendly one. May charge a bit more, but yeah, he’s the one to go to.” And knowing the right people, and sometimes it almost feels like that old boys’ network … whereas somebody else, who may not be plugged into that English-speaking professional class or social context, or … may just be using different social media apps, or just interacting with different groups on social media, just would not have this information.’*	002, Representative from an organisation who supports the general LGBTQI+ community in Singapore
Treatment of LGBTQI+ minors being dependent on parent’s preferences for care	*‘I think it’s, there’s an additional layer of complexity when it comes to youth, especially the legal minors. So because the parents ultimately have control over the health care of the kids … what kind of medical help they can seek, especially if we’re talking, if we’re talking about mental health issues. It really heavily depends on what the parent wants for the kid.’*	005, Representative from an LGBTQI+ brand serving the LGBTQI+ community

For individuals with intersectional identities – such as being an ethnic minority, gender-diverse, young or old, or with a history of substance use – stigma effects overlapped in unique forms of disadvantage.

Being transgender or otherwise gender-diverse was frequently mentioned throughout majority of interviews as an LGBTQI+ identity that especially disadvantaged individuals within Singaporean healthcare, for various reasons: healthcare administrative systems often follow binaries in which transgender individuals do not fit well; gender-affirming care is neither subsidised nor conveniently accessible in Singapore; and research and knowledge about the transgender population is lacking locally.

### Recommendations to reduce stigma in healthcare and beyond

Our interviewees shared recommendations for policy (both healthcare-specific and otherwise) and healthcare education, depicted in Table 7. With regards to healthcare training, an interesting recommendation from multiple respondents was that LGBTQI+ health content should be integrated more subtly and holistically into local medical education, rather than as a stand-alone module or topic. Emphasis was also placed on community involvement in the creation of curricular content. Instead of aspiring towards depth of knowledge in LGBTQI+ health, interviewees quite unanimously echoed that foundations were essential: sensitivity, empathy, and professionalism in keeping personal views and clinical care separate. Several respondents also raised the importance of establishing safe spaces – whether for patients at public healthcare institutions, or for queer-affirming healthcare providers to come together, network and improve the queer-affirming healthcare scene locally.

**Table 7.** Sub-theme 5: *Recommendations to reduce stigma in healthcare and beyond.*

Subtheme	Quote	Interview reference number
Recommendations regarding general socio-legal environment in Singapore	*‘I think a lot of it has to come from: what kind of examples are you setting? … How is it institutionalised? So obviously, official policies need to be changed, that’s the most straightforward and impactful way you can improve stigma. If [national media regulation authority] decides that they won’t hate gay people or queer people anymore, then it will make a huge difference in terms of media representation, in terms of the discussions, in terms of people just being able to see themselves on the TV … for young people who are growing up, it makes a huge difference to the future mental like, well being.’*	005, Representative from an LGBTQI+ brand serving the LGBTQI+ community
Recommendations regarding healthcare-specific policy	*‘One thing that I do in terms of my work at [redacted – restructured hospital centre where interviewee works] is: We have to come up with guidelines … when we talk about pap smears, we would say that a pap smear is applicable to people with vaginas, instead of saying that all females should do a pap smear, because that can be quite transphobic.’* *‘There are still community mental health providers. But that’s very few … it would be good if our government hospitals and all that can actually set out these safe spaces.’’*	009, Representative from an organisation supporting the general LGBTQI+ community in Singapore 003, Representative from an organisation which addresses sexual health in Singapore
Suggestions for content in medical education	*‘I think [for] a lot of healthcare professionals, training comes from folks who don’t actually know the community. So I think it’s more like working with a community to get them to provide that kind of training’* *‘Having basic gender sensitivity … understanding how to be a supportive ally … learning to be patient with your patient … using the right name, using the right pronouns … just [a] general level of sensitivity. The basics [are] important.’*	012, Representative from a community group advocating for the bisexual population in Singapore 007, Representative from an organisation supporting the transgender community in Singapore
Suggestions for delivering LGBTQI+ related health content in medical education subtly	*‘I don’t know if introducing a LGBT health-specific module in medical school is going to help … that might be putting a square peg in a round hole, because it may not be fitting what we need. I wonder if a more … “sneaky” way might be helpful. I mean, talking about communication skills, start there–…using more nuanced language … more open, gender-neutral language … not jumping into. assuming, before your patient tells you what their needs are.’*	002, Representative from an organisation supporting the general LGBTQI+ community in Singapore

## Discussion

Our findings suggest stigma is present within healthcare in Singapore towards LGBTQI+ patients. This can impede their access to local healthcare services, compromise healthcare received, and result in negative physical and mental health sequelae. Our findings also suggest that part of the stigma within healthcare may be due to a lack of training – as evidenced by sub-optimal knowledge and self-reported preparedness scores among a representative sample of local clinical year medical students about LGBTQI+ health. Simultaneously, however, this same pool of clinical year medical students reported moderately high levels of comfort and perceived importance towards LGBTQI+ health, as well as moderately positive attitudes. This study is the first within Singapore to formally establish and qualify the presence of healthcare-related stigma towards LGBTQI+ people, and to explore the perceptions and knowledge levels of local medical students towards LGBTQI+ health. These findings provide an evidence base to support the development of and guide future interventions to reduce stigma and improve medical professionals’ capabilities in caring for LGBTQI+ patients.

The findings from our interviews with respect to healthcare stigma towards LGBTQI+ patients mirror those of other studies internationally: health inequalities for LGBTQI+ patients manifest due to an undercurrent of heterosexism and discrimination, across levels ranging from healthcare institutions to the wider community [43–46]. In addition, our interviews highlighted the role of intersectionality in constructing unique forms of layered discrimination for certain more vulnerable LGBTQI+ patients, as has been previously highlighted in Singapore among young queer men [18]. In particular, having a history of substance use, being young or old, and being an ethnic minority all emerged as key identities which compounded the effects of stigma on LGBTQI+ patients. This is particularly the case in Singapore where community groups have reported how substance use is tightly intertwined with the criminal justice system [47], which may hinder treatment-seeking behaviour among those who use substances [48,49]. LGBTQI+ issues, too, are divisive across generational lines in Singapore, with many regarding LGBTQI+ priorities as affecting the young [50]. This may result in older LGBTQI+ individuals feeling outcast, avoiding approaching or being out to their healthcare providers. Conversely, this may result in young LGBTQI+ individuals’ experiences and needs being poorly accepted by their parents who may gatekeep their access to health [51]. In Singapore, too, most of the population is ethnically Chinese [52] and able to speak English and/or Mandarin. Individuals who are ethnically Malay or Indian and predominantly Malay- or Tamil-speaking do make up the population too, and would thus experience structural barriers to care if queer-affirming services are primarily English or Mandarin-based. In addressing stigma within LGBTQI+ health in Singapore, it would be especially pertinent to cater to how intersectionality manifests within our local context.

Similarly, the findings from our surveys with respect to medical students’ perceptions towards LGBTQI+ health are consistent with international studies. In a study in New England, over half of the 658 medical students surveyed reported inadequate preparation to serve LGBTQI+ patients; the overall self-reported confidence levels to treat LGBTQI+ patients were limited while overall self-reported comfort levels were moderate [24]. In a study of 103 medical students in Canada, most were comfortable with providing medical care to LGBTQI+ patients [22]. Unlike in our sample, though, most students felt capable of providing medical care to LGBTQI+ patients [22]. In the Malaysian study examining attitudes among medical students towards LGBTQI+ patients, the mean attitudes index scores using the ATLPS-M were similarly moderately high as in our sample [25]. Similarly to our sample, two-thirds of medical students in a study in the United States rated their medical school’s LGBT-related curriculum as ‘fair’ or worse [21].

Putting these findings together, our study team has several recommendations. On a more systemic and societal level, cues can be taken from international practice with respect to laws and policies governing LGBTQI+ individuals. In Singapore, besides the recent repeal of Section 377A, which criminalised consensual sex between men [53], the interwoven rights of LGBTQI+ individuals can be better ensured. This can include access to housing, employment, media representation, and legal protection from discrimination. Understanding the social determinants of health, such socio-economic protections can trickle down to benefit health and wellbeing [54]. These steps, though foundational, are crucial to LGBTQI+ people within Singapore, considering the complex socio-cultural milieu of the country and the range of prevailing attitudes towards the LGBTQI+ population [55]. Healthcare-specific laws and policies should also be addressed to treat members of the LGBTQI+ community more equitably and equally, such as laws and processes for gender marker change [56,57], or improved safeguards to ensure non-discriminatory medical practice [46].

On a more micro-level, we recommend curricular enhancements to improve the competency of healthcare workers to care for the LGBTQI+ population. Targeted training of healthcare students and providers has been shown to improve knowledge, attitudes and practice with regards to LGBTQI-specific healthcare in the short term [27]. Certain universities have already developed certificate programmes in LGBTQI+ health as well [58]; some have programmes centering the social determinants of health [59]; while others have successfully employed simulation in LGBTQI+ healthcare training [60]. As mentioned in our interviews, the literature also recommends that marginalised communities themselves should be involved in medical education efforts related to reducing health inequities [61]. In Singapore, this could mean collaborations between medical schools and the key local LGBTQI+ organisations or community members in developing medical education programmes. Such training may also build more allyship within the healthcare professional community. Allyship was not only associated with better scores in comfort and attitudes towards LGBTQI+ health in our survey, but newfound allies can in turn advocate for the above-mentioned systemic recommendations to be implemented on a wider scale too [62].

### Limitations

This study is not without limitations. Firstly, the qualitative component comprised interviews with representatives of LGBTQI+ organisations, rather than of a larger sample of LGBTQI+ individuals themselves; some narratives experienced by LGBTQI+ individuals themselves within Singapore may have not been captured. Nevertheless, representatives were able to articulate the experiences of their beneficiaries, clients and/or community members. Secondly, certain subpopulations of the LGBTQI+ community were less well-represented than others – such as the Singaporean intersex community. Thirdly, the quantitative component measured preparedness through self-reported data. Preparedness for LGBTQI+ health may be more precisely assessed through observer ratings in interactions with LGBTQI+ standardised patients – and this serves as a possible follow-up study. Lastly, this study assesses the adequacy of curricular coverage of LGBTQI+ content in medical schools via students’ perceptions; a more objective future endeavour would be to review curricular coverage systematically.

## Conclusion

The effect of stigma on health among LGBTQI+ patients in Singapore is well-felt among the community. With favourable attitudes and comfort levels among medical students, there is great potential in future curricular intervention. Taken together, the findings of this mixed methods study are a call to action for wider policy in Singapore, its healthcare institutions, and for its medical education system.
